# Supplementary material for: Chemotherapy use and outcomes in patients with stage III or IV small-cell lung cancer in relation to age: An analysis of the English Systemic Anti-Cancer Treatment (SACT) dataset
Source: Open Res Eur. 2023 Feb 20;3:35. [Version 1] doi: 10.12688/openreseurope.15602.1 (PMC11216366; doi:10.12688/openreseurope.15602.1)
Supplement: Peer review history

# Reviewer report: Reviewer response for version 1

**DOI:** 10.21956/openreseurope.16862.r41418

**Published:** 2024-06-29

**Authors:** Charles Gaber

**Affiliations:** 1 University of Illinois Chicago, Chicago, Illinois, USA

In this publication, Pilleron et al. describe the chemotherapy use patterns and survival outcomes of a cohort of nearly 8,000 patients diagnosed with Small Cell Lung Cancer (SCLC) in the United Kingdom (U.K.) between 2014-2017. The time period of analysis is noted to be prior to the immunotherapy era for SCLC in the U.K. The Systemic Anti-Cancer Treatment (SACT) dataset was used to identify this cohort. The authors report on chemotherapy dose reductions, delays in the administration of chemotherapy regimen, and stopping the planned regimen early. In terms of survival outcomes, the authors report 30 and 90 day mortality rates along with 6 and 12-month overall survival and median survival. All measures are jointly stratified by stage of cancer (III or IV) and age (<75 or ≥75).

 The authors find that older patients were substantially less likely to receive curative treatment compared to younger patients in both stages (Stage III: 19.8% versus 3.3% and Stage IV: 7.2% versus 0.8%). Older adults were more likely to experience dose reductions than younger patients, but, interestingly, this finding was confined to the Stage III sub-cohort and not observed in the Stage IV sub-cohort. Perhaps unsurprisingly, survival was lower in the adults ≥75 years of age and those with Stage IV disease. The 12-month survival ranged from about 55% in those <75 with Stage III cancers to about 18% in those ≥75 with Stage IV cancers.

 This descriptive observational study is well-designed and executed. The SACT dataset is population-based and comprehensive in reach (in the U.K.) which mitigates concerns about an unrepresentative population (i.e., the sample in this study is the target population). The outcome measures, which are not measurable in many other real-world data sources, are well described and the statistical analyses are appropriate for a descriptive epidemiologic study. Lastly, the conclusions are placed in context with the existing literature end emphasize the importance of the findings even in the immunotherapy era; many patients in the U.K. still receive chemotherapy alone (possibly due to comorbidities or contraindications) and the numbers reported in the study can inform care discussions about prognosis.

Is the study design appropriate and does the work have academic merit?

Yes

Is the work clearly and accurately presented and does it cite the current literature?

Yes

If applicable, is the statistical analysis and its interpretation appropriate?

Yes

Are all the source data underlying the results available to ensure full reproducibility?

Yes

Are the conclusions drawn adequately supported by the results?

Yes

Are sufficient details of methods and analysis provided to allow replication by others?

Yes

Reviewer Expertise:

Cancer pharmacoepidemiology; geriatric oncology health outcomes research

I confirm that I have read this submission and believe that I have an appropriate level of expertise to confirm that it is of an acceptable scientific standard.

# Reviewer report: Reviewer response for version 1

**DOI:** 10.21956/openreseurope.16862.r39923

**Published:** 2024-05-06

**Authors:** Anke Richters

**Affiliations:** 1 Netherlands Comprehensive Cancer Organisation, Nijmegen, The Netherlands

This is a well-written manuscript that provides clinically relevant data on the use of chemotherapy among patients with SCLC, particularly focusing on older patients. This manuscript contributes to optimal treatment selection especially in older patients and is based on seemingly strong data source that is appropriate for good descriptive analyses.

Is the study design appropriate and does the work have academic merit?

Yes

Is the work clearly and accurately presented and does it cite the current literature?

Yes

If applicable, is the statistical analysis and its interpretation appropriate?

Yes

Are all the source data underlying the results available to ensure full reproducibility?

Yes

Are the conclusions drawn adequately supported by the results?

Yes

Are sufficient details of methods and analysis provided to allow replication by others?

Yes

Reviewer Expertise:

Cancer epidemiologist

I confirm that I have read this submission and believe that I have an appropriate level of expertise to confirm that it is of an acceptable scientific standard.
